# Root-filling materials for endodontic surgery: biological and clinical aspects

**DOI:** 10.2340/biid.v11.42172

**Published:** 2024-10-29

**Authors:** Andreas Koutroulis, Vasileios Kapralos, Dag Ørstavik, Pia Titterud Sunde

**Affiliations:** a Section of Endodontics, Institute of Clinical Dentistry, Faculty of Dentistry, University of Oslo, Oslo, Norway; b Division of Endodontics, Clinic of Conservative and Preventive Dentistry, Center for Dental Medicine, University of Zurich, Zurich, Switzerland

**Keywords:** Endodontic surgery, retrograde root filling materials, biological properties

## Abstract

The placement of root filling materials aims to prevent the occurrence of post-treatment apical periodontitis following completion of endodontic treatment. Materials should possess properties that will not permit bacterial invasion and infection, namely excellent sealing ability and/or antibacterial properties. In root-end filling procedures or repair of root perforations, the root filling materials are placed in a particularly challenging clinical environment, as they interface with a relatively large area with the periradicular tissues. The biological properties of these materials are therefore of significant importance. The current review discusses the most widely used materials for endodontic surgery (i.e., root-end filling and perforation repair), with particular focus on their biological characteristics, namely antibacterial properties and interactions with host tissue cells, together with clinical studies. Properties of amalgam, glass ionomer cements (GICs), resin systems, zinc oxide eugenol-based cements and hydraulic calcium silicate cements (HCSCs), together with representative and well-researched commercial materials in the context of their use in endodontic surgery are presented. While the use of HCSCs seems to offer several biological advantages, together with addressing issues with the initial formulation in the most recent versions, materials with different chemical compositions, such as zinc oxide eugenol-based cements, are still in use and appear to provide similar clinical success rates to HCSCs. Thus, the significance of the currently available materials on clinical outcomes remains unclear.

## Introduction

The cause-and-effect relationship between bacteria and pulpal or periapical diseases highlights the primary aim of clinical endodontics: treating and/or preventing microbial contamination [1]. The placement of root filling materials is the last step of all these clinical protocols, with the main goal to seal the root canal system from periradicular tissues in order to prevent bacterial invasion and infection after completing the treatment [2]. Technically, root filling materials can achieve this task by means of physical integrity and excellent adaptation to dentin walls or by exerting antimicrobial activity [3].

In nonsurgical root canal treatment, an inert core material (gutta-percha) is combined with an endodontic sealer. The core material acts as a piston for the sealer, which fills the gaps between the gutta-percha and dentin, and basically determines the functional characteristics of the root filling [4]. In surgical procedures and perforation repairs, a single endodontic material is used [5].

The exposed material surface in contact with the periradicular tissues in conventional root filling procedures of mature teeth with closed apices is relatively small as this occurs mainly through the apical foramina and any lateral canals. In contrast, in clinical procedures entailing endodontic surgery, perforation repair or treatment of immature teeth with open apices, the contact area with the subsequent tissues may be considerably larger [3]. The clinical environment to which the root-end filling and perforation repair materials are exposed is inherently humid because of blood and other tissue fluids. In addition, the materials typically meet an inflamed tissue that has been formed in response to the preceding infection [6]. These materials therefore have a complex role towards blocking the pathways of communication of the root canal system with the periradicular area in a dynamic environment. Furthermore, it is deemed beneficial if they can facilitate healing of the periradicular tissues [7]. Materials are placed in direct contact with dentin and the periradicular tissues but interactions with the subsequent tissues may not be limited in direct contact, as leachable material elements can reach further beyond the application field [3]. The physical and chemical characteristics of the material will therefore define their biological profile.

From this point in the article, the term ‘materials for endodontic surgery’ will be used inclusively to refer both to root-end filling materials and those used for perforation repair, as the materials are used in the same clinical environment. Independent reference will be made towards specific use if relevant. It is recognised that repair of coronal perforations in certain cases might be also performed through the root canal instead of surgically [5], and thus a non-differentiating inclusion of perforation repair materials under the term ‘surgery’ is not strictly accurate. However, it will be used further in terms of convenience. The term ‘extra-radicular materials’ has been previously used in the same context [8] but might also lack accuracy as it implies that materials are located outside of the root.

Historically, a variety of materials with different chemical compositions has been used for endodontic surgery. These include gold foil and leaf, gutta-percha (both cold and injectable), teflon, titanium screws, amalgam, silver points, zinc oxide/calcium sulfate cements, polycarboxylate cement, polyvinyl resin, poly-HEMA (hydroxyethyl methacrylate), resin-based sealers, various zinc oxide/eugenol (ZOE)-based cements, glass ionomer cement (GIC), composite resin, and, most recently, hydraulic calcium silicate cements (HCSCs) [9–11].

The aim of the present narrative review is to provide an overview of the most widely used endodontic surgery materials, with a focus primarily on biological properties and clinical performance.

## Discussion

### Amalgam

Silver amalgam was for many years the most vastly applied material for endodontic surgery [12–14], while its use remained popular among dentists even until recently [15]. The main advantages of the material were the ease of manipulation, non-induction of resorption in the dentinal tissues and its adequate radiopacity [16]. Several drawbacks in regards to its properties have been reported in the literature, with the most significant being: concerns on its biocompatibility [17], which is associated with mercury release [18], corrosion [19], and tissue staining because of silver salts [20, 21]. Reports have also indicated that amalgam may be prone to leak, as demonstrated in laboratory assessments using dye penetration assays [22, 23], fluid filtration systems [24], or bacterial inocula [22, 25]. However, these experimental investigations have faced criticism for their lack of reproducibility and high variability in results [26], which limits their clinical implications and they are no longer recommended to be undertaken [27, 28].

Histological signs of extensive host tissue inflammation in response to amalgam application have been reported, which were more severe than ZOE-based cements, mineral trioxide aggregate (MTA) and a resin-modified GIC [29–32], with the exception of one study that found non-significant differences with a ZOE-based material [33]. Furthermore, the use of amalgam has been associated with poor prognosis [34, 35]. Apart from the available scientific evidence today on the superiority of other materials for endodontic surgery, the use of amalgam is additionally not recommended by the European Union because of environmental pollution concerns related to mercury release [36].

### Glass ionomer cements

Glass ionomer cements consist of an acid-decomposable fluoroaluminosilicate glass powder combined with a polyacrylic acid liquid [37]. Tartaric and maleic acids were additionally incorporated into the liquid component to act as modifiers of the setting reaction [38, 39]. Glass ionomer cements were introduced in the 1970s [40, 41] and have since gained popularity in clinical dentistry, particularly as restorative cements because of their adhesion to dentin via ionic exchange at the interface, and a caries-inhibitory potential through fluoride release [42]. In root-end filling procedures, the application of GICs does not necessitate mechanical cavity preparation, but it does require a dentin pre-treatment stage. The removal of the dentin smear layer is necessary before the application of GIC to enable adhesion [5].

Material properties primarily depend on the specific glass composition, the particle size, the powder/liquid ratio, and the concentration of the acid liquid [37, 43]. A common composition initially used in commercial GICs consists of SiO_2_, Al_2_O_3_, AlF_3_, CaF_2_, NaF, AlPO_4_ [44]. Since then, a plethora of compositions has been developed, including aluminium-free ones containing strontium [45] or zinc [46], as well as formulations that partially substitute calcium with magnesium or zinc [47], among other formulations.

*In vitro* cellular studies evaluating the cytocompatibility of GICs for potential use in endodontic surgery have found mild to severe cytotoxicity [48–50] and low cellular attachment to the material surface [51]. In addition, a study showed that GIC failed to preserve the integrity of periodontal tissues following its application in artificially induced perforations in dogs [52]. Conversely, another study reported acceptable cytocompatibility values following cellular exposure to GIC [53]. Intraosseous implantation of GIC resulted in mild initial inflammation, which gradually decreased over time [54].

The antibacterial properties of GICs primarily stem from their acidity [55, 56], while a potential association with increased fluoride release has also been noted [57], which is attributed to its inhibitory potential against bacterial enzymes [55]. Topical application of fluoride-containing toothpastes and gels led to a recharge of GICs, prolonging their antibacterial potential by fluoride release *in vitro* [57]. However, this does not have a practical application in the surgical endodontic field.

Regarding overall clinical performance, a systematic review of clinical studies concluded that GIC and amalgam exhibited similarly poor performance as root-end filling materials [58].

### Resin-modified GICs

A significant modification of GICs was performed by introducing a resin component in them. The addition of the light-cured polymer in resin-modified GICs improved several material characteristics, particularly the working and setting time, but it also significantly reduced the fluoride release [5, 43]. The materials are dual cure: light-activation in addition to the acid-base reaction taking place. The most popular representative of this material category in endodontic surgery is Geristore (Den-Mat, Lompoc, CA, USA). Interestingly, resin-modified GICs have shown good biocompatibility values *in vitro* [51, 59–63] and can therefore be considered for application in endodontic surgery. When used as a retrograde filling material in infected dog teeth, however, cases treated with Geristore showed signs of chronic inflammation histologically after 6 months, despite radiographic evidence indicating healing [64].

### Resin systems

Dentin bonding agents, along with the use of resin composite have found application in endodontic surgery. Adjusted from restorative dentistry to the specific surgical field, these systems still require good control of humidity during application [65, 66]. In the case of retrograde filling, a conservative cavity with a concave shape is prepared followed by etching of the dentin surfaces [67]. The primary advantages of these systems are bonding to dentin and the sealing of exposed dentinal tubules [66]. The best known material in this category is RetroPlast (RetroPlast Trading, Roervig, Denmark).

The release of unreacted monomers by the resin systems has been associated with cytotoxicity [5]. Studies of dentin bonding agents have reported a suppression in the normal macrophage function and an initial cytotoxic behaviour [68–70]. But studies on RetroPlast demonstrated contradictory findings with either a proliferative effect in contact with fibroblasts [62] or inhibition of the cell growth of fibroblasts and macrophages [71] and poor fibroblast attachment upon the material [51]. When implanted into the dorsal connective tissue of rats, resin systems induced a moderate to severe inflammation that declined within a 3-month observation period [72]. The antibacterial effect has been shown to vary among different commercial materials, depending on the inclusion of antibacterial monomers or fluoride in their composition [73].

Although several clinical studies have reported good outcomes from using the resin systems with the accompanied preparation technique [65, 74–76], a study found that the resin-system technique had a lower weighted pool success rate compared to the contemporary root-end surgery with non-resin cement [77]. This was mainly attributed to bonding failure because of the humid application field [77]. However, the effect of different materials was not assessed in the meta-analysis, and the outcomes seem to have been mainly influenced by one [78] of the three included studies using the resin-system technique.

### Zinc oxide/eugenol-based cements

Zinc oxide/eugenol cements have been a staple in endodontic surgery for many years [79, 80]. These cements are formed from zinc chelates with eugenol (zinc-eugenolate salt) in a relatively weak bond [3], and currently have two main modified commercial representatives: Super EBA (Bosworth, Skokie, IL, USA) and Intermediate restorative material (IRM; Dentsply Sirona, Charlotte, NC, USA). In Super EBA, eugenol is partly substituted by ethoxy benzoic acid (68%) in the liquid component and the addition of fumed silica and, later on, aluminium oxide (34%), along with natural resin (6%) in its powder formulation [81]. The IRM contains polymethacrylate (20%) within its powder composition, along with the addition of acetic acid (1%) to the liquid component [5, 82]. The adaptation of ZOE-based cements to dentin is achieved with purely mechanical means.

The biological reactivity of the ZOE-based cements is attributed to eugenol release by progressive hydrolysis of the material surface [83, 84], which might even increase over time [73]. Cytotoxicity assessments of Super EBA and IRM have yielded contradictory results when compared to amalgam [85–88]. Some *in vitro* studies reported cytotoxic effects of Super EBA on gingival fibroblasts and periodontal ligament cells [89–92], as well as in undiluted extracts against cancer cells [93]. Similarly, IRM exhibited the highest cytotoxicity on fibroblasts [71, 94, 95] and osteoblasts [87] among some contemporary materials used in endodontic surgery. Differences in cell culture systems and the criteria used for assessment could markedly affect the results of cell viability experiments [48].

In studies conducted on monkeys, Super EBA and IRM elicited a less severe inflammatory response in the periradicular area compared to conventional unmodified ZOE cement [30] and amalgam [30, 96, 97]. In another animal study involving dogs, IRM was found to be more effective than Super EBA in promoting the re-establishment of periradicular tissues [98]. Although one clinical animal study did not find any evidence of hard tissue formation within 5 weeks of applying IRM to healthy resected dog teeth [99], a subsequent study demonstrated that IRM promoted healing after 6 months when used as a retrograde material in infected dog teeth [64].

Only few studies have investigated the antibacterial properties of Super EBA and IRM with particular reference to their application in endodontic surgery. Some studies have evaluated the antibacterial properties of these materials by measuring zones of bacterial inhibition on agar medium [100–102], a method that is now considered less accurate because of its susceptibility to the materials’ diffusion in the agar, which can affect the results [27]. In direct contact test against planktonic bacteria, IRM exhibits antibacterial activity especially when freshly mixed, but the activity may persist in freshly set samples, lasting up to at least 1 day [103]. Another study found this effect extended to 3-day in set samples and included resistance against *Staphylococcus aureus* and *Pseudomonas aeruginosa* as well [73]. A moderate yet sustained antibacterial effect has been also reported, lasting even after 18 weeks of material storage in water [104]. In a dentin infection model, a decline in the antibacterial efficiency of IRM against *E. faecalis* was observed after 7 days [105]. Additionally, an assessment of minimum bactericidal concentration deemed IRM’s efficacy acceptable against *E. faecalis*, *S. aureus* and *Streptococcus mutans* [106].

Several human clinical studies have demonstrated that the ZOE-based cements exhibit acceptable success rates following the endodontic surgery [107–111], with follow-up evaluations performed up to 2 years for IRM [112] and up to 4 years for Super EBA [113, 114]. Thus, IRM and Super EBA continue to be recommended for use in endodontic surgery [115].

### Hydraulic calcium silicate cements

Hydraulic calcium silicate cements are used today in various endodontic procedures [5, 116, 117]. The term ‘hydraulic’ is derived from the Greek word hydra, meaning ‘water’, and is used to denote that water is the reactant for setting of these materials [118]. ‘Calcium silicate’ is used to indicate the materials’ basic chemical composition, with their primary constituent being tricalcium silicate [119]. To be classified as an HCSC, the hydration reaction must be the primary reaction that occurs [119], leading to the formation of calcium silicate hydrate and calcium hydroxide. While several other terms have been assigned to this material category, the current one appears to be the most appropriate for adequately specifying their chemistry and main attributes [119].

An abundance of clinically used HCSCs is available today. These may be classified on two levels: (1) based on their composition, and particularly on the main components that can modify their hydration (Table 1); (2) based on the endodontic procedure they are indicated for (i.e. ‘intra-coronal, intra-radicular, extra-radicular’) [8].

**Table 1:** Classification of commercially available hydraulic calcium silicate cements based on cement type, presence of additives, and water component (i.e., liquid vehicle) [8].

Type	Cement	Additives	Liquid vehicle	Representative material
1	Portland cement	−	√	ProRoot MTA (Dentsply)
2	Portland cement	√	√	NeoMTA Plus (Avalon Biomed)
3	Portland cement	√	−	Bio-C repair (Angelus)
4	Tricalcium/dicalcium silicate	√	√	Biodentine (Septodont)
5	Tricalcium/dicalcium silicate	√	−	TotalFill (FKG Dentaire)

### Type 1 HCSCs – Mineral trioxide aggregate

More than a century after the first reported use of Portland cement to fill root canals [120], Torabinejad patented a new material for ‘*tooth filling*’ under the name MTA [121]. The MTA is a blend of Portland cement (80%) with bismuth oxide radiopacifier (20%), mixed with distilled water [122] (Type 1 HCSC).

Portland cement is commonly used in the construction industry as a binder for concrete, and is composed of tricalcium silicate (45–70%), dicalcium silicate (5–30%), tricalcium aluminate (<10%), calcium aluminoferrite (<10%), and trace oxides (0.5%) from raw materials. Calcium sulfate is added in the final step [122, 123]. When mixed with water, the following reactions occur:

- Tricalcium aluminate dissolves and reacts with calcium and sulfate ions. Calcium aluminate sulfate is formed and precipitates upon the cement surface.
- The hydration reaction of (tri-, di-) calcium silicates produces amorphous calcium silicate hydrate and calcium hydroxide.

The MTA shares the properties of Portland cement and incorporates bismuth oxide to provide radiopacity. After a series of *in vitro* investigations [86, 100, 124], animal studies [125–128] and clinical applications in patients [129], MTA was commercialised as ProRoot MTA (Dentsply, Tulsa Dental, Johnson City, TN, USA) in 1998.

ProRoot MTA is a type 1 HCSC. A white version (tooth-coloured ProRoot MTA) became available a few years later with a significantly lower concentration of iron [130], in an effort to address concerns about the induction of tooth discoloration. Soon, other companies began to launch their own ‘MTA’s, with first being Angelus (Londrina, Brazil) in 2001. Today, several commercial products have the term MTA in their name. Despite some adjustments from the initial composition in some of the most recent products and occasional confusions created by the commercial exploitation of ‘MTA’ as a trade name, the term corresponds to mixtures of Portland cement-like materials with a chemical compound as radiopacifier (type 1 or 2 HCSCs) [5]. In the current overview, the term MTA is used to refer both to the ProRoot and Angelus versions. Type 3 materials could be considered as MTA-like, since they are Portland cement-based HCSCs but with the main difference being that they are not mixed with any liquid vehicle (Table 1) and their hydration relies solely in the reaction with the environment fluids [5].

The MTA powder is hand spatulated with distilled water, resulting in a grainy mixture that can be challenging to apply [123]. The setting process can extend for several hours as the initial stages of the reaction of Portland cement have a complex sequence of procedures [123]. The exclusion of calcium sulfate from the cement, which has been conducted in the MTA Angelus, accelerates the reaction [131, 132]. Following material hydration, the cement structure consists of a matrix of amorphous calcium silicate hydrate interspersed with non-hydrated cement particles and bismuth oxide particles [133]. Calcium hydroxide leaches out and interacts with the local environment [134], substantially raising the pH [124, 135, 136]. Bismuth oxide was found not to remain inert; it interferes with the hydration of tricalcium silicate [133], leaches out from the cement [134] causing tooth discoloration [137] and has been shown to be cytotoxic [138, 139]. Recently, it has been replaced by other radiopacifiers in some formulations, namely calcium tungstate in MTA Angelus [140].

The biological properties of MTA have been extensively assessed using various methods, from cellular studies to research involving animals and humans. Overall, it appears that the material is cytocompatible [91–93, 95, 141–150]. Fresh specimens might induce an initial cytotoxic effect but this is followed by cell recovery [48, 71, 88, 94], or even enhancement of cell growth in aged samples [90, 145]. However, cumulative material leaching *in vitro* from 1 to 42 days resulted in a decrease in cell survival [151]. The absence of cytotoxicity of MTA is further supported by the enhancement of cellular attachment on its surface with osteoblasts [87, 152–154], bone marrow mesenchymal stem cells and periodontal ligament stem cells [155]. Additionally, it appears that MTA enhances the secretion of biochemical markers associated with new bone formation [145, 148, 156, 157] and modulates the secretion of inflammatory mediators, contributing to the resolution of inflammation and tissue repair [146, 152–154].

Tissue reactions have been histologically assessed following subcutaneous or intraosseous implantation of MTA in animals. Studies indicate an inflammatory reaction of varying severity during the initial days of implantation, which typically subsides over time [158–169], and may as well be accompanied by tissue repair and healing [170]. Notably, when dentin blocks filled with MTA were implanted in the dorsal tissue of rats, mineral deposition in the material/dentin interface occurred [171]. This apatite precipitation upon the material surface coincides with the initial inflammatory phase [172], and suggests that MTA promotes a beneficial proinflammatory and pro-wound environment [171, 172]. Moreover, the use of MTA as root-end filling material induced soft and hard tissue formation in the periapical tissues of both healthy and infected dog teeth [64, 98, 99, 173]. In cases of perforations treated with MTA, an initial inflammatory response was observed after 1 week, which subsided at 1 month [174], while other studies observed healing with hard tissue-like formation after 3 [175, 176] or 4 [177–179] months.

The antimicrobial potential of MTA, as in all HCSCs, stems mainly from the alkaline-induced environment [180, 181]. Its antimicrobial effect is strong in fresh samples and appears to decrease in aged specimens [73, 182–185]. However, two studies demonstrated that MTA exhibited stable antibacterial behaviour between 30 min and 24 h of set samples in direct contact with planktonic *E. faecalis* [186] or between 24 h and 7 days of set samples in a dentin infection model against biofilms of the same species [105]. The presence of dentin substrate in the latter study was hypothesised to enhance its effect [105]. Overall, the antibacterial effect seems to depend on the bacterial species being tested [73], with susceptibility varying also among different strains [186]. Another study reported ‘*acceptable*’ values of minimum bactericidal effect against *S. aureus, E. faecalis* and *S. mutans*, yet the results were not compared to any control or were not assessed statistically [106]. Against multispecies biofilms, MTA failed to show any potent activity [187]. The somewhat contradictory nature of data from different studies can be attributed to varying assessment methods, including direct contact tests and dentin infection models [187, 188]. In a laboratory study using conditions relevant to endodontic surgery, exposure to blood was found to neutralise MTA’s antibacterial activity [184].

In human clinical studies, the use of MTA for root-end filling has demonstrated similar success rates to IRM [109, 112] and Super EBA [111, 114]. Utilising microsurgical techniques in conjunction with MTA-application yields high success rates [189–192]. The clinical success of MTA for perforation repair has been investigated scantly [193], but, as explained above, the conditions are practically the same.

### Types 4 and 5 HCSCs

As Portland cement is manufactured by naturally occurring raw materials, concerns have been raised about the presence of trace metal elements in MTA [194, 195], most importantly aluminium ions, which have been found to leach into peripheral organs [196, 197], and have even been associated with the induction of oxidative stress in the brain [198]. These considerations led to the replacement of Portland cement by pure calcium silicates manufactured using laboratory-grade materials [199, 200] (types 4 and 5 HCSCs). The difference between type 4 and 5 HCSCs is the absence of a liquid component in the latter, which use a water-free vehicle to suspend the powder instead [5, 201]. The inclusion of additives in the calcium silicate phase of the materials as well as in the liquid component of type 4 HCSCs is carried out mainly to improve issues with the handling characteristics and long setting time of MTA. Two representative and well-researched materials from these categories are: Biodentine (Septodont, Saint Maur-des-Fosses, France) and TotalFill BC (FKG Dentaire, La Chaux-de-Fonds, Switzerland) formulations (Table 1).

### Representative type 4 HCSC

Biodentine, classified as a type 4 HCSC, was originally introduced as a dentin replacement material, but it is also indicated for endodontic surgery [202] and commonly used for perforation repair [115]. The powder phase of the material consists of tri- and di-calcium silicate (80%), calcium carbonate (15%), iron oxide (<1%), and zirconium oxide (5%) [200]. Calcium carbonate acts as an accelerator of the hydration reaction [203]. The material exhibits lower radiopacity than MTA [200], which can be attributed to the relatively low amount of zirconium oxide radiopacifier. The liquid component includes water, calcium chloride as a setting accelerator and a water-soluble polymer to enhance workability and reduce the water/powder ratio [200, 204]. The material powder is supplied in a capsule and is mixed with the recommended powder/liquid ratio in a triturator. Biodentine has a setting time of approximately 12 min [202], which is considerably reduced compared to MTA [203], while it also presents enhanced mechanical properties compared to MTA [204].

Given that MTA preceded type 4 and 5 HCSCs by several years, it routinely serves as a reference material for evaluating the biocompatibility of newer formulations. These comparisons have provided varying results. Biodentine’s 24-h extracts had a negligible cytotoxic effect on primary human osteoblast, similar to MTA [151]. Furthermore, while the cumulative 42-day extract of MTA induced cytotoxicity, Biodentine’s effect was not significantly altered in that period [151]. No cytotoxicity was induced in human periodontal ligament cells by 3-day extracts of Biodentine, which reported values similar to MTA [205]. On the contrary, other studies found that neat 24-h extracts of Biodentine induced cytotoxicity in apical papilla cells [147], murine mesenchymal stem cells [156] and osteoblasts [206], while eluates of MTA or MTA-like materials performed better [156, 206] or even did not affect cell viability at all [147]. In a wound healing assay, MTA allowed for unhindered cellular migration and proliferation, in contrast to Biodentine, but both cements showed adequate cell attachment upon their surfaces [147]. Direct exposure to a fibroblast cell line showed overall similar values of cell viability for Biodentine and MTA compared to the control, with significantly more cells adhering to the Biodentine surface than MTA [207]. In regard to the osteogenic potential, Biodentine stimulated differentiation of murine mesenchymal stem cells similar to MTA [156] as well as enhanced the mineralisation activity in osteoblasts [206, 208, 209]. In an *ex vivo* model assessing the regeneration of bone defects in mice, Biodentine eluates had a slightly lower potential compared to MTA [148]. In another study, Biodentine was as effective as MTA in suppressing inflammatory markers [209].

Subcutaneous implantation of Biodentine in rats induced a moderate inflammation at 7 days, which subsided by 14 days [160]. In a dog periradicular surgery model, both MTA and Biodentine induced periradicular healing [205]. Furthermore, Biodentine and MTA promoted similar healing responses when used to treat furcation perforations in rats, with reduction in inflammation from 14 to 21 days and decreased bone resorption [210]. At 60 days, both materials led to narrowed periodontal spaces, reduced numbers of immune cells and osteoclasts, and increased densities of osteoblasts, fibroblasts, and collagen [211]. In dog models with artificially induced furcation perforations, Biodentine and MTA showed overall similar outcomes: one study reported comparable hard tissue formation for both materials but noted a lower degree of inflammation induced by Biodentine [179], whereas other studies found that MTA exhibited a better mineralisation potential [178] and resulted in smaller proportions of inflammatory scores [174].

The evaluation of the antimicrobial activity of Biodentine has yielded reports of varying efficiency against different bacterial species and in comparison to MTA [212–215]. Several studies have used cariogenic bacteria, which might not be relevant in the context of endodontic surgery, but they do provide information on the material’s overall antibacterial potential. Using a direct contact test, Biodentine and MTA exhibited similar antibacterial efficiency against *E. faecalis*, with the effect decreasing when samples aged [182]. In another study, incubation of Biodentine with a planktonic *E. faecalis* culture yielded bacterial growth after 24 h exposure [216]. In studies with cariogenic bacteria, fresh Biodentine was highly effective against a planktonic polymicrobial *Streptococcus* suspension upon direct contact, but the effect decreased with longer exposure, allowing eventually biofilm formation upon its surface [217]. The material leachate had no effect on the same suspension [218]. Biodentine exhibited antibacterial activity against *S. mutans* both in direct contact [219] and leachate assays [220], as well as a moderate effect against *Lactobacillus casei* [220]. However, it had no impact on a multispecies biofilm in a dentin model [187].

Human clinical studies on the use of Biodentine in endodontic surgery are limited, possibly because it is primarily used for perforation repair rather than root-end surgery [115]. Its clinical potential for root-end filling has been suggested in case reports [221, 222]. In a clinical case trial with artificially induced perforations in teeth scheduled for extraction and assessed histologically after 3 months, Biodentine showed comparable biocompatibility to MTA, but the latter showed more formation of cementum-like tissue [223].

### Representative type 5 HCSC

The term ‘root repair material’ abbreviated as ‘RRM’ will be utilised consistently throughout the remainder of the article to refer to a group of materials that are marketed under various commercial names, such as TotalFill (FKG Dentaire, La Chaux-de-Fonds, Switzerland), Endosequence (Brasseler, Savannah GA, USA), and iRoot (Veriodent, Vancouver, Canada), possibly because of regional licensing issues. These ‘*premixed*’ materials (type 5 HCSCs) do not contain a liquid component and undergo thus, hydration in the presence of environmental fluids, similar to type 3 HCSCs (Table 1). A range of materials with different consistencies is available, which were specifically introduced for root repair. These include RRM paste, RRM putty, and RRM fast set putty. The composition of these formulations is rather similar, as it is also denoted by the fact that they have the same safety data sheet [224].

The manufacturer reports a composition of tricalcium silicate (30–36%), dicalcium silicate (9–13%), calcium sulfate (3–8%) and radiopacifiers zirconium oxide (15–18%), and tantalum pentoxide (12–15%) [224]. Additionally, the materials contain a calcium phosphate phase, which, although not explicitly mentioned in the safety data sheet, is documented in the ‘*Instructions for use*’ file for Endosequence BC RRM [225]. The presence of two radiopacifiers renders the materials particularly radiopaque [226]. As regards their setting times, one study reported an initial setting time of 62 min for the RRM paste and 18 min for the RRM fast set putty [227]. Another study found initial setting times of 2 h for the RRM paste and 22.5 h for the RRM putty [226]. These variations can be attributed to differences in testing conditions, including immersion in water in the first study and exposure to 95% humidity in the second [226].

In terms of biological properties, exposure of an osteoblast cell line to RRM putty resulted in higher numbers of viable cells and lower rates of apoptosis compared to MTA and RRM paste, which were also cytocompatible [228]. The 7-day extracts of MTA and RRM putty had a similar effect on cell viability of human gingival fibroblasts, but the 2-day RRM putty extracts were more cytotoxic [92]. Similar cell viability values (> 90%), were also obtained with 24-h leachates on dermal fibroblasts, although the putty scored lower values than the negative control [142]. Another study found that the 2- and 7-day extracts of RRM putty and RRM paste had similar cell viability values to MTA, except for the 2-day RRM paste, which was more cytotoxic compared to MTA [95]. All three materials promoted cell attachment [95, 155, 228]. RRM putty exhibited potent osteogenic behaviour with increased expression of osteoblastic genes, mineralisation activity and acceptable cell viability in both an osteoblast cell line and rat mesenchymal stem cells, but in the case of the latter, MTA had an overall superior effect [149, 157].

Comparison of healing outcomes between RRM paste and MTA in root-end surgeries on dog teeth with apical periodontitis showed no significant difference in the inflammatory infiltration and cortical plate healing 6 months after the operation, but the RRM had more favourable tissue healing adjacent to the resected root-end surface [173]. In artificially induced perforations in rat teeth, after an initial inflammatory response at 1 week, the RRM putty induced complete cortical plate healing at 1 month, whereas in 50% of the Biodentine cases, the periodontal ligament had still necrotic areas infiltrated by inflammatory cells [229].

The RRM paste and putty have demonstrated comparable antimicrobial efficacy to MTA in a direct contact test against *E. faecalis* and *Candida albicans*: fresh and 1-day set samples reduced the microbial colonies, although this effect diminished in the 7-day aged samples [185]. In another study, all three materials remained equally antibacterial after 7 days against different *E. faecalis* strains [186]. When assessed against cariogenic bacteria, Biodentine and RRM formulations have shown similar efficacy [219, 220].

Human clinical studies on the use of RRM putty in endodontic microsurgery have reported similarly favourable outcomes as those observed with MTA in terms of clinical and radiographic examination for at least 1 year of follow-up [190–192].

## Conclusions

The use of HCSCs in endodontic surgery today gathers several advantages over other contemporary used materials. The first cement, MTA, introduced for endodontic surgical applications, has undergone extensive laboratory and clinical research, which has enhanced our understanding in the basic chemistry of this material. Furthermore, the replacement of the Portland cement with tricalcium silicate cement and the substitution of bismuth oxide with inert radiopacifiers in MTA have been supported by substantial laboratory and clinical evidence in materials that have been in use for over a decade now [230]. At the same time, a concern in this perspective is that new commercial formulations of HCSCs are constantly emerging in the dental field, claiming modifications that often lack comprehensive research prior to clinical application or even full manufacturer documentation.

Overall, it is important to emphasise that despite the advances in material development, especially since the introduction of HCSCs, the significance of the currently available materials on clinical outcome remains unclear [78, 231]. This also raises the question of whether materials like IRM, which has several decades of clinical application but lacks the biological interaction of HCSCs, will/should eventually be replaced. Therefore, the role of material selection in the clinical outcome of endodontic surgery requires further assessment (232).

## Data Availability

Data sharing is not applicable to this article as no new data were created or analysed in this study.

## Key messages

- Over the past years, a variety of materials with different chemical composition has been employed for use in endodontic surgical procedures.
- Hydraulic calcium silicate cements gather several advantages in terms of biological properties over other material categories.
- Despite the superiority of hydraulic calcium silicate cements, further scientific evidence is needed in order to determine the effect of material selection on the clinical outcomes of endodontic surgical procedures.
